# A Non-genotoxic Variant of *Escherichia coli* Nissle 1917 EcN 2.0 Overexpressing Microcins Reduces Intestinal Carriage of ST131 ESBL-Producing *Escherichia coli*

**DOI:** 10.1007/s12602-025-10777-y

**Published:** 2025-11-11

**Authors:** Nicolas Jousserand, Benjamin Massiera, Pierre-Jean Bordignon, Michelle Boury, Marie Tremblay-Franco, Ulrich Dobrindt, Patricia Martin, Rachel Lavoué, Eric Oswald, Delphine Payros

**Affiliations:** 1 IRSD, Université de Toulouse, INSERM, INRAE, ENVT, UPS, Toulouse, France; 2 Toxalim (Research Centre in Food Toxicology), Université de Toulouse, INRAE, ENVT, INP-Purpan, UPS, Toulouse, France; 3 Institute of Hygiene, University of Münster, Münster, Germany; 4 CHU de Toulouse, Hôpital Purpan, Toulouse, France; 5 Department of Anesthesiology and Critical Care Medicine, University Hospital of Toulouse, University Toulouse III-Paul-Sabatier, Toulouse, France; 6 Present Address: National Vet School of Toulouse (ENVT), INRAE, UMR1225, IHAP, Université de Toulouse, 31300 Toulouse, France

**Keywords:** Multidrug-resistant *Escherichia coli*, Intestinal carriage, Biotherapeutic strategy, Probiotic Nissle 1917, Microcin, Colibactin, Alternative to antibiotic

## Abstract

*Escherichia coli* (*E. coli*) ST131 is a multidrug-resistant pandemic clone of extended-spectrum beta-lactamase-producing bacteria, with the primary reservoir within the gut. A biotherapeutic strategy to limit the intestinal carriage of ST131 *E. coli* isolates has been developed using a modified version of the probiotic *E. coli* strain Nissle 1917 (EcN 2.0) that does not exhibit genotoxic activity and overexpresses microcins. The capacity of EcN 2.0 to inhibit in vitro the growth of *E. coli* was demonstrated using strain JJ1886, a representative human clinical ST131 isolate. We evaluated the efficacy of EcN 2.0 in a murine model of stable colonization of the intestinal microbiota by the multidrug-resistant *E. coli* JJ1886. The administration of EcN 2.0 reduced the individual load of JJ1886 in feces when compared to the placebo-treated animals. Following a 30-day course of daily oral administration of EcN 2.0, 8 of 17 mice no longer excreted the ST131 strain. Furthermore, the results demonstrated that EcN 2.0 was more efficient than EcN WT against clinical multidrug-resistant uropathogenic *E. coli* isolates from humans and animals. Accordingly, the probiotic EcN 2.0 may represent a viable strategy to limit the carriage of ST131 and potentially other multidrug-resistant pandemic enterobacterial clones, which in turn could help limit the spread of antimicrobial resistance and the incidence of urinary tract infection.

**Supplementary Information:**

The online version contains supplementary material available at 10.1007/s12602-025-10777-y.

## Introduction

Antimicrobial resistance is a main public health concern that is increasingly impacting the medical management of infectious diseases in humans and pets. Many strains can produce extended-spectrum beta-lactamase (ESBL) enzymes that provide resistance to most currently used beta-lactam antibiotics, frequently recommended as treatment for bacterial diseases such as urinary tract infection in human and veterinary medicine [1, 2]. These strains are also frequently resistant to fluoroquinolones and represent the most common cause of multi-resistant bacterial infections and extra-intestinal *E. coli* infections [3]. The origin of the spread of antimicrobial resistance is multifactorial, and the *E. coli* clone ST131 is the globally dominant multi-resistant representative of extraintestinal pathogenic *E. coli* [3]. JJ1886 is a well-documented strain belonging to ST131 lineage, isolated from a woman with fatal urosepsis in the USA [4]. This strain is affiliated with the H30-Rx sub-lineage, due to its carriage of CTX-M-15 beta-lactamase-encoding gene (Fig. S1). The emergence and dissemination of the ST131 clone may relate to its capacity to efficiently colonize the gut in an asymptomatic manner. So, the gastrointestinal tract is considered the main reservoir of ST131 *E. coli* strains [5]. Thanks to their persistence in the gut and under specific stress conditions, immune suppression, or increase of inflammation status, these strains can translocate from the intestine, spread to other niches, and be responsible for extra-intestinal infections such as sepsis or urinary tract infections.

As the combined and sequential use of multiple antimicrobials leads to the emergence of increasing levels of resistance, several approaches have been considered to reduce the intestinal carriage of ESBL-producing *E. coli*. Bacteriophage cocktails have been widely investigated and demonstrated promising experimental results [6, 7]. Similarly, administration using probiotic *E. coli* or other bacterial species has been successfully reported, as preventive or therapeutic treatment [8–11]. However, recently bacteriophages and probiotics have been used together and displayed preventive action for intestinal colonization in mice [12].

Among probiotic strains, *E. coli* Nissle 1917 (EcN) is commercially available as “Mutaflor” and has been described as a prophylactic and therapeutic treatment for acute or chronic inflammatory bowel disease, particularly for ulcerative colitis [13–15]. EcN has been demonstrated to have health benefits such as anti-inflammatory activity or induction of digestive immune defenses [16, 17]. EcN has been previously described as capable of having antimicrobial activity against several other members of the *Enterobacteriaceae* [18–20], notably through the production of siderophore-microcins. Compared to conventional antibiotic treatment, microcins act at a short distance on a narrow spectrum of bacteria, and therefore have only a minimal effect on the gut microbiota and the emergence of antimicrobial resistance [21, 22]. We hypothesize that EcN is capable of eliminating multidrug-resistant *Enterobacteriaceae* within the gut reservoir, thus limiting their carriage.

Although the EcN strain is presented as non-pathogenic, it carries a genetic island coding for colibactin, a genotoxin that causes DNA double-strand breaks and chromosomal aberrations in eukaryotic cells [23, 24] and suspected to play a role in colorectal cancer [25]. Recent data has shown that colibactin can induce the SOS response in bacteria [26] and may therefore be a factor that contributes to antibiotic resistance [27, 28]. Thus, the safety of EcN needs to be discussed [29].

The objective of our research was to develop a non-genotoxic version of EcN that would allow us to capitalize on its established probiotic attributes while mitigating the potential risks associated with the synthesis of active colibactin. In addition, we sought to improve the production level of microcins, creating the EcN 2.0. Subsequently, the efficacy of the non-genotoxic EcN strain EcN *clbH*^−^ and EcN 2.0 in controlling the intestinal carriage of ESBL-producing enterobacteria was assessed. It was postulated that an EcN variant that over-expresses microcins would be capable of eradicating *E. coli* ST131 within the gastrointestinal reservoir, thereby limiting its excretion in feces and subsequent dissemination in the environment.

## Materials and Methods

### Bacterial Strains, Mutants, and Plasmid

Bacterial strains, plasmid, primers, and oligos used in this study are summarized in Table S1.

**Fig. 1:**
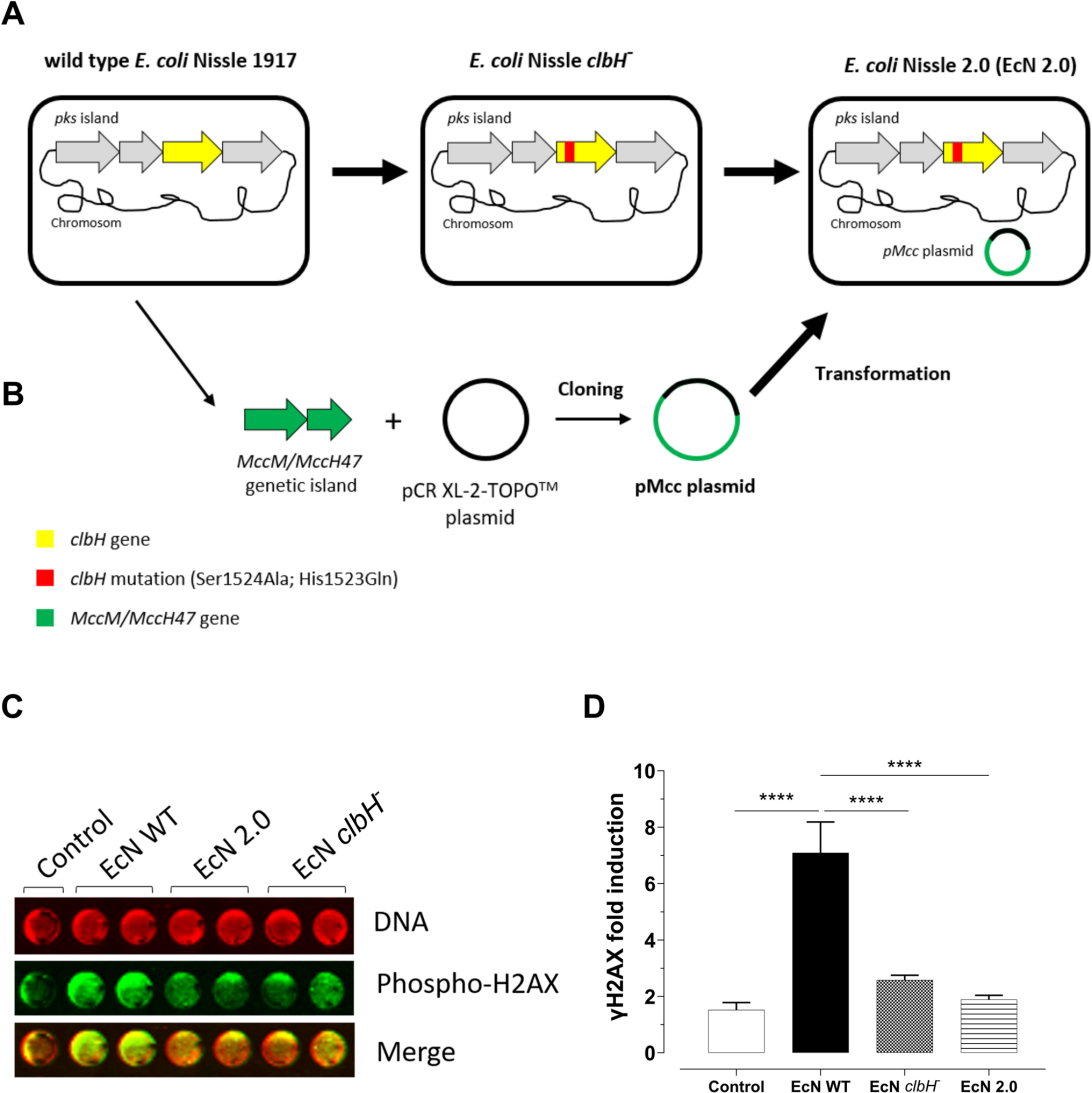
Construction of non-genotoxic version of *E. coli* Nissle 1917 overexpressing siderophore-microcins: EcN 2.0. (A, B) Schematic representation of construction of EcN 2.0. (A) Chromosomal *clbH* gene was mutated in wild-type strain of EcN with pORTMAGE technique. EcN was transformed with the pORTMAGE plasmid and the lambda-red system carried by the plasmid was induced by heat shock and the bacteria rendered electrocompetent. EcN was contacted with a specific 90-nucleotide oligo (Supplementary Fig. 1A), designed to inactivate the catalytic site of the enzyme encoded by the *clbH* gene (Ser1524Ala;His1523Gln). After electroporation, EcN was grown under specific conditions and the cycle repeated 3 times. (B) MccM/MccH47 genetic island of EcN WT (4.8 kb) was cloned and inserted in a plasmid TOPO-XL. To ensure the inability of the EcN 2.0 strains to produce colibactin, we quantified the phosphorylation of histone H2AX (γH2AX), a marker of DNA double-strand breaks in eukaryotic cells. (C) ICW pictures of non-infected (control) HeLa cells or infected with Nissle *E. coli* strains producing (EcN WT) or not producing colibactin (EcN 2.0 or EcN *clbH*^−^) at multiplicity of infection of one hundred. At the end of the interaction, cells were washed and incubated 4 h with gentamicin containing complete medium culture before ICW staining. DNA is stained in red, γH2AX in green. (D) Quantification of γH2AX in HeLa cells infected for 4 h with EcN WT, EcN 2.0, and EcN *clbH*^−^ or not infected with *E. coli* (control) at multiplicity of infection one hundred. *N* = 4 independent experiments. Mean values ± standard deviation are shown. One-way ANOVA with Tukey’s multiple comparison post-test. **** *p* < 0.0001

The EcN *clbH* chromosomal isogenic mutant was constructed using the Multiplex Automated Genome Engineering (MAGE)–based mutagenesis plasmid pORTMAGE as previously published [30]. The protocol followed was similar to that used in the work of Massip and collaborators [31]. Briefly, the pORTMAGE plasmid produces a dominant negative mutant protein of the methyl-directed mismatch repair system and is used to transiently suppress DNA repair, which is necessary for efficient oligonucleotide integration. After EcN wild-type transformation with pORTMAGE, by three iterative cycles, combinatorial genetic diversity was generated. A 90-nucleotides oligonucleotide was used for inactivation of serine from the catalytic site of *clbH*^−^ encoded enzyme (Fig. 1(A)). EcN/pORTMAGE was grown in LB with antibiotic at 34 °C at 300 rpm until bacteria reached an OD_600nm_ of 0.6. λ-red genes were induced by heat shock (42 °C, 250 rpm) for 15 min and were then quickly transferred on ice. The bacteria were pelleted by centrifugation and washed twice with ice-cold water. EcN bacteria were mixed with a specific oligo and electroporated (Table S1). For recovery, the bacteria were incubated at 34 °C for 3 h. This cycle was repeated three times. The resulting bacterial suspension was plated on LB agar and 96 isolates were subcultured and stocked. Two different experiments were performed to generate two independent series of 96 mutants. With them, growth curves (OD_600nm_) in rich (Lysogeny Broth) and poor (M63) medium were recorded to identify specific isolates presenting the expected mutation in the *clbH* gene in comparison to EcN WT (data not shown). Afterwards, the *clbH* mutation and the loss of pORTMAGE was verified by whole-genome sequencing: paired-end sequencing, 2 × 150 bp, on an Illumina NovaSeq 6000 platform (Integragen Genomics®, Evry, France). BioNumerics® software (Applied Maths NV®) was used for analysis. To overexpress the siderophore-microcins M and H47, the 4.8-kb genetic island MccM/MccH47 was cloned and inserted in a plasmid TOPO-XL that carries the machinery required for the microcin biosynthesis and export (named pMcc, Fig. S2). The EcN *clbH*^−^ strain selected previously was transformed with pMcc. Successful transformants were selected on LB agar plates supplemented with kanamycin. The resulting strain was named *E. coli* Nissle 2.0 (EcN 2.0) (Fig. 1(B)).

For later screening applications, the ESBL-producing *E. coli* ST131 strain JJ1886 was tagged with a chromosomal and permanently expressed resistance marker. Due to the high number of antimicrobial resistances naturally present in *E. coli* JJ1886 strain (Fig. S1), rifampicin was chosen as the identification antimicrobial molecule. *E. coli* JJ1886 was cultured for 24 h in four mL lysogeny broth (LB) with 25 µg/mL of rifampicin at 37 °C and 240 rpm. Subsequently, this culture was propagated for 5 days by daily serial passage of 1/100 in fresh LB with 50, 75, 100, 150, and 200 ug/mL of rifampicin. The next day, 10 clones were isolated from LB supplemented with 100 µg/mL rifampicin and 100 µg/mL carbenicillin and sequenced.

### Collection of Animal and Human Clinical Strains

Human clinical strains come from a bank of more than 200 *E. coli* isolates collected from patients with bacteriuria (asymptomatic bacteriuria, acute cystitis, and pyelonephritis) at the University Hospital of Toulouse [32]. In accordance with French regulations on the analysis of observational databases, no specific informed consent was required for the collection of clinical *E. coli*. For animal strains, similarly, strains come from a bank of 150 *E. coli* isolates collected from dogs and cats at the Veterinary Teaching Hospitals of the Toulouse and Alfort Veterinary Schools [33].

### Determination of the Genotoxic Effect of EcN Strains

To investigate the genotoxic activity of EcN strains, HeLa cells (ATCC CCL2) were cultivated in 96-well cell culture plates (1.5 × 10^4^ cells/well) with DMEM + 10% Fetal Calf Serum (FCS) + 1% non-essential amino acids (Invitrogen) supplemented with 100 µg/mL gentamicin in 37 °C 5% CO_2_ incubator. Twenty-four hours later, the medium was replaced by a DMEM-25 mM HEPES medium (Invitrogen) after three times PBS washing. Cells were infected with EcN strains with a multiplicity of infection of 100 bacteria per cell. After 4 h of incubation (37 °C, 5% CO_2_), the cells were washed three to five times with warm PBS and the medium was replaced by DMEM + 10% FCS + 1% non-essential amino acids supplemented with 200 µg/mL gentamicin. Cells were maintained for a duration of 4 h post-infection. The medium was removed and after three flushes with PBS, the cells were fixed with 4% formaldehyde for 20 min. After three washing steps with PBS, an In-cell Western analysis was performed as previously described [34]. Briefly, the fixed cells were permeabilized with 0.2% Triton X-100 for 15 min and incubated in Maxblock solution (1 h) before staining with anti-phospho-H2AX (rabbit monoclonal antibody, 20E3, Cell Signaling, Saint-Quentin en Yvelynes, France, 1:200) for 4 h. After washing, near-infrared-fluorescent secondary antibody (IRDye 800 CW; Rockland, 1:500) and RedDot2, a marker for DNA staining (Biotium, Interchim, Montluçon, France, 1:1000), were used for 1 h. The phospho-H2AX and DNA signals were measured at 800 nm and 680 nm, respectively, with a Sapphire Biomolecular Imager (Azure Biosystems). The phospho-H2AX fold-induction was calculated by dividing the phospho-H2AX fluorescence by the corresponding DNA fluorescence and normalized with the average fluorescence in untreated control cells [35].

### Competition in Liquid and Solid Medium Assay

*E. coli *microcin-emitting strains (EcN WT, EcN *clbH*^−^, and EcN 2.0) or not (*E. coli* K-12 strain MG1655 strain was used as negative control) and the target strain JJ1886 were grown separately in LB for 18 h at 37 °C with the required antibiotic (streptomycin 50 µg/mL, kanamycin 50 µg/mL, rifampicin 200 µg/mL) at 240 rpm. In 50-mL tubes, 10 mL of M63 minimal medium was inoculated with a 1/20 dilution of the culture of each strain and cultured at 37 °C and 240 rpm to obtain bacteria at the end of the exponential growth phase (around 2 h of culture). M63 iron was composed of 1 g/L Bacto tryptone (BD Biosciences), 15 mM ammonium sulfate, 1 mM magnesium sulfate heptahydrate, 100 mM monopotassium phosphate, 2.5 g/L glucose, and 1 mg/L thiamine.

For liquid competition assays, the OD_600nm_ was obtained for each strain and 10^6^ CFU of a microcin-emitting strain (or PBS as control) was added to 10^6^ CFU of the target strain in 10 mL of M63 minimal medium in 50-mL tubes. The tubes were incubated at 37 °C and 240 rpm. After 24 h, serial 10 to 10 dilutions in PBS were plated on LB agar plates supplemented with 50 mg/mL rifampicin. After 24 h of incubation at 37 °C, the number of colonies was counted.

For the competition assay on solid medium, the OD_600nm_ was adjusted to adjust the bacterial concentration to 5.10^8^ CFU/mL. Five hundred microliters of a 1/100 dilution was spread on M63 minimal medium + 1% agar and striated over the entire plate. After a few minutes of drying, a drop of 2.5 µL of the preculture of each microcin-emitting strain was deposited in pre-defined areas. Plates were incubated at 37 °C for 48 h. The diameter of the inhibition halo was measured for each microcin-emitting strain.

### Experimental Animal Model

All the experimental procedures were carried in accordance with the European directives for the care and use of animals for research purposes and were validated by the local ethics committee from CREFRE US006 (Regional Centre for the Functional Exploration and Experimental Resources) (number 21-U1220-EO/NJ-268).

Certified pregnant Swiss mice (Janvier Labs, Le Genest Saint Isle, France) received streptomycin in their drinking water until they gave birth. At birth, antibiotic treatment stopped. The neonates and the nest are brushed with 10^e^9 bacteria from *E. coli* JJ1886. The animals were maintained without intervention for 21 days until weaning. Biweekly quantification of intestinal carriage of *E. coli* JJ1886 was performed using fecal samples from eight litters. Three litters were excluded due to insufficient or heterogeneous colonization, and the remaining five litters—showing consistent colonization levels over time and between cages—were retained for analysis. Animals from these litters were evenly distributed between the control and EcN 2.0 treatment groups (see Fig. S3 and Table S2).

At 5 weeks, the animals were divided into two homogeneous groups (50% females, 50% males, from four different litters). The control group received 100 µL of 1 × PBS daily and the group treated with EcN 2.0 received 10^9^ CFU/mL in 100µL of 1 × PBS by oral force-feeding for 1 month (31 days). At the end of the experiment, feces were collected for the enumeration of JJ1886 and performing enrichment for the detection of *E. coli* JJ1886. To better assess the host response to EcN 2.0 treatment, we included an evaluation of clinical and macroscopic parameters in all animals at the end of the experiment. These analyses encompassed body weight gain, colon length and thickness, and a macroscopic score after dissection (Table S3).

### Determination of the Fecal Bacterial Load

The colonic bacterial load in feces was analyzed before oral force-feeding with EcN strains or PBS to ensure the level of colonization by the target strain JJ1886. After the beginning of treatment, quantification of intestinal carriage of *E. coli* JJ1886 was performed biweekly in the feces of animals upon completion of the experiment.

As previously described [36], feces homogenates were prepared and tenfold serial dilution was plated on MacConkey agar plates supplemented with appropriate antibiotics (rifampicin) and incubated overnight at 37 °C. The numbers of CFU were enumerated after 18 h. At the end of the experiment, feces were collected and frozen in nitrogen before storage at − 80 °C. The feces were cultured for 24 h in liquid MacConkey medium supplemented with rifampicin to select the strain of interest JJ1886. The next day, the growth of the bacteria was assessed in the rifampicin-containing medium to determine the clearance of animals for the strain JJ1886.

### Statistical Analysis

Statistical analyses were carried out using GraphPad Prism 8.0 (GraphPad, San Diego, CA, USA). Mean values ± SEM are shown. *P* values were calculated using one-way ANOVA followed by Tukey’s multiple comparison post-tests. CFU/g of feces and CFU/mL were log-transformed for the analyses. In Fig. 4, analyses were performed with R software (www.r-project.org). The two-tailed signed-rank Wilcoxon test was used to determine the change in colonic bacterial load in feces during the study. This nonparametric paired test enabled the comparison of log CFU values between the two groups (PBS/EcN *clbH*^−^ or PBS/EcN 2.0) and within the groups (before treatment = *t* 0 and after 31 days). In Fig. 5, bacterial shedding of *E. coli* JJ1886 in mice was evaluated by the log-rank (Mantel-Cox) test.

## Results

### Construction of Non-genotoxic Version of *E. coli* Nissle 1917 Overexpressing Siderophore-Microcins: EcN 2.0

As mentioned in the introduction, EcN produces colibactin, a genotoxin. To avoid its cytogenotoxic effect before using it as a probiotic strain, the *clbH* gene was inactivated, because the non-ribosomal peptide synthetase ClbH is directly responsible for the addition of a cyclopropane (C_3_H_3_) group, which mediates DNA alkylation, to precolibactin [37]. The isogenic colibactin-negative mutant EcN *clbH*^−^ was constructed using Multiplex Automated Genome Engineering (MAGE)–based mutagenesis targeting the *pks* island with pORTMAGE as previously published [30] (Fig. 1(A)).

The competitiveness of EcN also depends on the production of siderophore-microcins [31]. Indeed, EcN is able to produce two types of microcins, i.e., microcin H47 and microcin M. They are encoded by a genomic island present on the EcN chromosome. To avoid limitations due to the regulation of expression of this genomic island, a plasmid was built, called pMcc, using TOPO™ XL by cloning the entire EcN MccM/MccH47 island. EcN *clbH*^−^ was transformed with pMcc to obtain a new non-genotoxic probiotic strain overexpressing siderophore-microcins, designated EcN 2.0 (Fig. 1(B)).

The genotoxic activity of EcN 2.0 was assessed by measuring the phosphorylation of H2AX, γH2AX, a marker for DNA double-strand damage in HeLa cells, and compared with the EcN WT strain. As previously shown in the laboratory, a direct contact between eukaryotic cells and live colibactin-producing *E. coli* strain is required to observe DNA damage (Nougayrède et al., 2006; [29, 38]). The results showed that in cells infected with the EcN WT strain, an increase of γH2AX signal was observed, resulting from colibactine-induced damage, but not the *pks* island–negative K-12 strain MG1655 used as a control. The *clbH*^−^ negative strains EcN *clbH*^−^ and EcN 2.0 did not induce γH2AX, indicating a loss of genotoxicity for these two strains on eukaryotic cells (Fig. 1(C, D)). Moreover, induction of genotoxicity by the colibactin-producing EcN strain did not result in cell detachment, as confirmed by the RedDot2 signal (Fig. 1(B)) and consistent with previously reported thresholds (Garofalo *et al.*, 2023), indicating that the infection did not cause substantial cell death.

### Overexpressing Siderophore-Microcins in EcN 2.0 Increases Competitiveness Against ESBL-Producing Pathogenic *E. coli* In Vitro

Next, the efficacy of EcN 2.0 overexpressing siderophore-microcins to outcompete the target ST131 *E. coli* strain JJ1886 was evaluated in vitro. The ESBL-producing uropathogenic *E. coli* strain JJ1886 belonging to the phylogroup B2 and sequence type ST131 was used as a model multidrug-resistant ExPEC strain (Fig. S1). In our study, we used a spontaneous mutant of *E. coli* JJ1886 resistant to rifampicin, which was validated by antimicrobial susceptibility testing and whole-genome sequencing (data not shown).

**Fig. 2:**
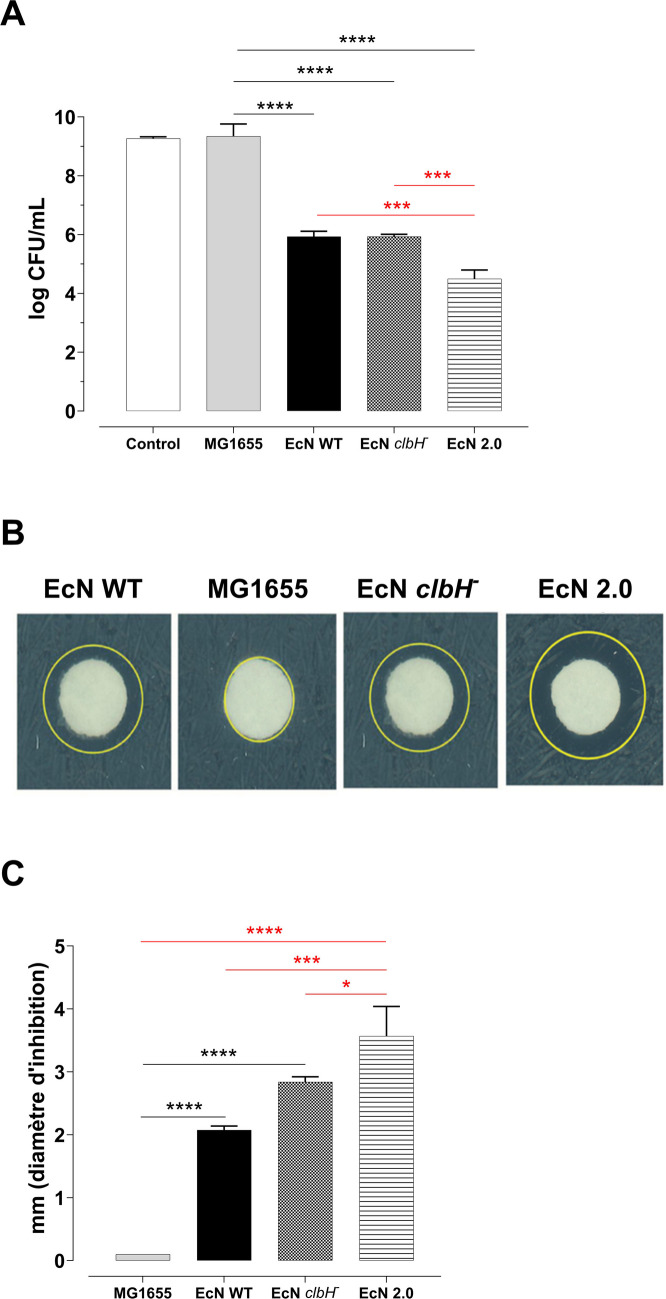
Evaluation of the antimicrobial activity of EcN 2.0 compared with EcN WT and EcN *clbH*^−^ against strain ST131 strain JJ1886. **A.** Competition in liquid medium. Target strain JJ1886 was grown alone or in combination with an antimicrobially active strain, EcN WT, EcN *clbH*^−^, or EcN 2.0 or a negative control (*E. coli* strain MG1655). After 24 h of incubation at 37 °C with agitation, the target strain was enumerated by successive dilutions and spread on antibiotic-enriched agar to select it.** B** Photos of growth inhibition during solid-state competition: the background of the image is a mat of ST131 *E. coli* JJ1886 target bacteria, the antimicrobially active bacterium was deposited on the blotting paper in between the image. The growth inhibition halo (yellow circle) was measured to quantify the inhibition.** C** Solid-state competition. Inoculum of 10^8^ colony-forming units (CFU) of target strain JJ1886 was plated on M63 agar. EcN or MG1655 bacteria (negative control) were plated on blotting paper. Growth inhibition of the target strain was quantified by measuring the inhibition halo formed around the plate. *N* = three independent biological replicates. Mean values ± standard deviation are shown. One-way ANOVA with Tukey’s multiple comparison post-test. In black, comparison between negative control MG1655 and EcN WT or EcN *clbH*^−^.*p < 0.05; ***p < 0.001; ****p < 0.0001. In red, comparison between EcN 2.0 and other experimental conditions. *p < 0.05; ***p < 0.001; ****p < 0.0001

At first, to ensure that the *clbH* deletion has no effect on the competitiveness of EcN, we compared in vitro the ability of EcN *clbH*^*−*^ to outcompete *E. coli* JJ1886 with that of the EcN WT strain as previously described [31]. We performed liquid co-culture experiments with strains EcN WT, EcN *clbH*^−^, and EcN 2.0 and with MG1655 as a negative control. In liquid co-culture, the growth of *E. coli* JJ1886 was strongly and significantly inhibited by both EcN WT and EcN *clbH*^−^ at similar levels (*p* < 0.0001, Fig. 2A). Overexpression of siderophore-microcins in EcN 2.0 further inhibited the growth of *E. coli* JJ1886 as compared to EcN WT and EcN *clbH*^−^ (*p* < 0.0001, Fig. 2A).

To complete the evaluation of the antimicrobial efficacy of EcN 2.0, we used a competition assay on solid medium. Briefly, a sensitive bacterial strain is inoculated on a low-iron agar. Blotting paper pellets were soaked in a solution containing the bacteria to be tested and placed on the low-iron agar, as in an antibiotic susceptibility test. The inhibition halos formed around the EcN WT and EcN *clbH*^−^ depositions were comparable in size on a lawn of *E. coli* JJ1886 mat, while its growth was not inhibited around the deposition of strain MG1655 (negative control, *p* < 0.0001, Fig. 2B, C). Moreover, EcN 2.0 generated a larger halo compared to both EcN WT and EcN *clbH*^−^ (*p* < 0.0001 and *p* < 0.05 respectively, Fig. 2B, C).

These results confirmed the higher activity of EcN 2.0 compared to EcN WT to limit the growth of the target *E. coli* strain JJ1886 without a negative effect of the mutation on the *clbH* gene.

### The ESBL-Producing *E. coli* Strain JJ1886 Is Transmitted from the Mother to the Offspring and Persistently Colonizes the Gut at Adulthood

To study the effect of treatment on the carriage of MDR bacteria in the intestine, common animal experimental models used often antibiotic treatment to allow intestinal colonization by relevant bacteria, which seems inadequate to study the carriage of multidrug-resistant strains and their propagation [12, 39, 40]. Indeed, in these models, the antibiotic treatment will create a dysbiotic state and an artificial niche in the intestine to allow the introduction of the bacterium of interest by drastically reducing the diversity of the microbiota.

**Fig. 3:**
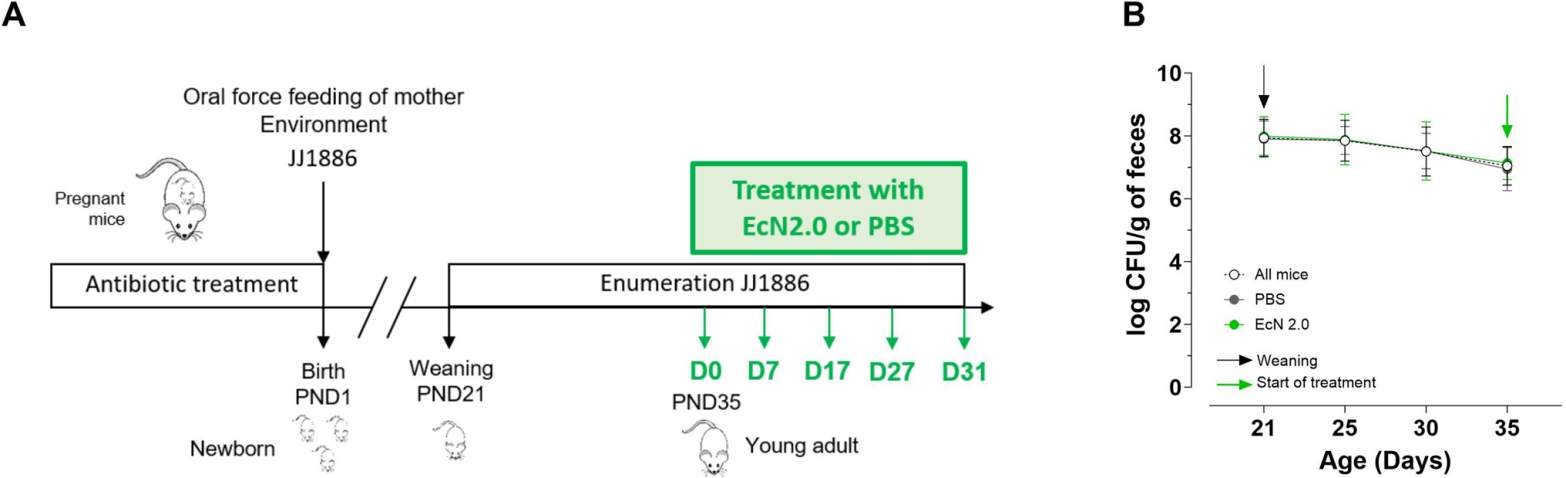
ESBL-producing *E. coli* JJ1886 strain are transmitted from mother to the offspring and persistently colonized the gut at adulthood without need antibiotic administration. **A** Experimental design of a mice mimicking long-lasting colonization by ESBL-producing *E. coli* JJ1886. Pregnant mice received streptomycin (5 g/L) in drinking water 3 days before parturition. At birth, mothers were inoculated with 10^9^ CFU ESBL-*E. coli* JJ1886 by spontaneous ingestion. JJ1886 were also added into the closest environment of the pups (nest) to favor the colonization of the gut. At weaning at post-natal day (PND) 21, gut colonization by JJ1886 was evaluated in feces homogenates until the end of experiment. At day 35 after birth, mice were stably colonized by JJ1886 and received daily 10^9^ CFU EcN 2.0 (probiotic treatment) or PBS (control) during 31 days (1 month) by oral gavage. At the end of the experiment, mice were euthanized. **B** Long-term carriage of ESBL-producing *E. coli* strain JJ1886 without need to administrate antibiotic treatment after birth at time points PND21 to PND35. Weaning is highlighted with a black arrow. The beginning of probiotic (EcN 2.0) or control (PBS) treatment is highlighted with a green arrow. Curves represented all the mice included in the experiment (white circle, dotted line) or the mice included in the groups treated with PBS (gray circles and line) or EcN 2.0 (green circles and line) showing the same basal level of colonization at the beginning of treatment (green arrow). Mean values ± standard deviation are shown for 32 mice from 4 different mothers

So, in this work, we used a physiological establishment model of multi-resistant enterobacteria to study the efficacy of EcN 2.0 treatment on the carriage of ESBL-producing ExPEC strains in the intestine (Fig. 3A). Inspired by a vertical transmission model already described by Payros and colleagues [41], pregnant Swiss mice received antibiotic treatment prior to delivery. At birth, antibiotic treatment was stopped and dams were fed with 10^e^9 CFU of the target strain JJ1886 diluted in PBS supplemented with 20% fructose, and newborns were brushed with 10^e^9CFU of the target strain JJ1886 diluted in PBS supplemented with 20% fructose in their closest environment (nest and litter). At post-natal day 21 (PND21) (weaning) until PND35, *E. coli* JJ1886 fecal bacterial load was quantified twice a week.

As the microbiota is established according to a precise chronology from birth, this model mimics its establishment in rodents with the first colonization by enterobacteria a few hours after birth. These bacteria come from the close environment of the newborn or the mother’s own microbiota. The individual bacterial load of *E. coli* JJ1886 was 10^e^8 CFU per gram of feces at PND21 and remained stable over time until PND35 in all animals, allowing us to assess the effect of EcN treatment on the carriage of EBSL-producing multi-resistant enterobacteria (Fig. 3B, Fig. S3. and Table S2.).

### Treatment with EcN 2.0, but not with EcN clbH− Reduced Shedding of ESBL-Producing *E. coli* ST131 JJ1886 in Mice

**Fig. 4:**
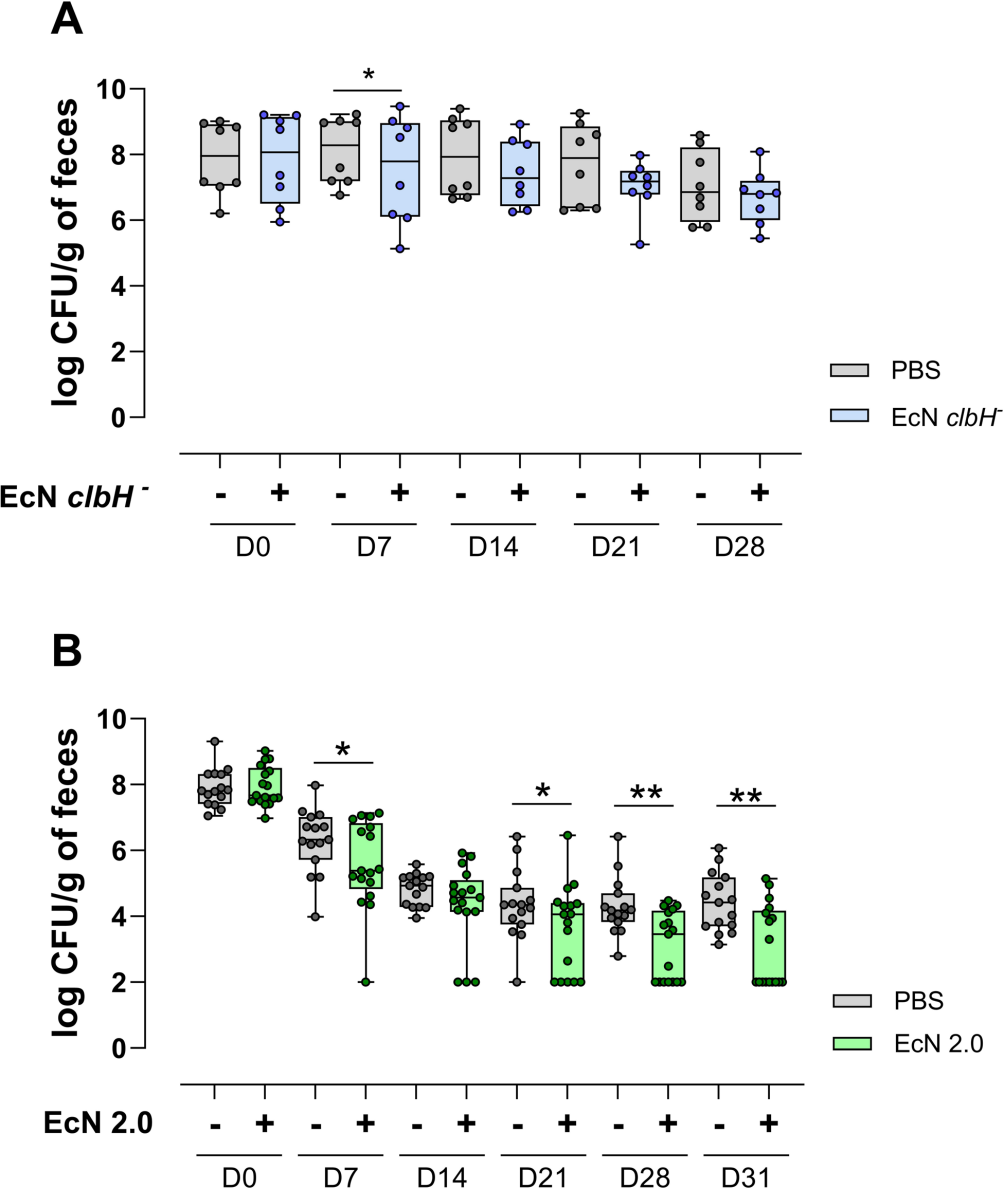
Bacterial shedding of JJ1886 in mice. Global evaluation of gut JJ1886 bacterial shedding in fecal homogenates in treated animals with EcN *clbH*^−^ (**A**, blue box and whiskers), EcN 2.0 (**B**, green box and whiskers) or not (grey box and whiskers) with EcN probiotic strains by oral force-feeding during 1 month. Each circle represents a mouse. Median are shown with black (vehicle) or blue or green (treated animals) line. Detection threshold = 2 log CFU/g of feces. Box and whiskers presenting median values ± min and max are shown. Each circle represents a mouse. *N* = 15 to 17 mice per group. Two-tailed signed-rank Wilcoxon test was used with nonparametric paired test between PBS and EcN *clbH*^−^ or PBS and EcN 2.0. *p < 0.05; **p < 0.01

To evaluate the efficacy of oral administration of probiotic strain EcN in reducing the intestinal carriage of the ESBL-producing strain JJ1886 based on our in vitro results (Fig. 2), two independent experiments were done on mice to test EcN *clbH*^−^ (Fig. 4A) and EcN 2.0 (Fig. 4B), respectively. Mice were distributed in two groups to ensure median *E. coli* JJ1886 loads were equivalent in each group (Fig. 3B). Mice received daily an oral gavage of 10^9^ CFU of EcN *clbH*^−^ (Fig. 4A) and EcN 2.0 (Fig. 4B) diluted in PBS or PBS alone for 1 month. The individual bacterial shedding of *E. coli* JJ1886 was monitored by feces collection once a week during the treatment period (Fig. 3A). Initially, the mean in the control group before treatment with EcN 2.0 or PBS was 7.14 log CFU/g feces and 6.94 log CFU/g feces, respectively.

No significant difference (*p*-value of the signed Wilcoxon test > 0.05) was observed between EcN *clbH*^−^-treated *versus* PBS group (Fig. 4A). An overall reduction in bacterial excretion was observed in animals treated with EcN 2.0 compared with animals given placebo (PBS group) (Fig. 4B). The median JJ1886 load in feces was significantly lower in mice treated with EcN 2.0 compared to control at days 7, 21, 28, and 31 (signed Wilcoxon test’s *p* < 0.05 and *p* < 0.01 respectively, Fig. 4B). For 8/17 animals in the group treated with EcN 2.0, the bacterial load was below the detection threshold (2 log CFU/gram of feces) at the end of the experiment. These results confirmed in vitro results and the efficacy of EcN 2.0 compared to EcN *clbH*^−^ to partially outcompete an ESBL-producing ExPEC strain in the murine long-term colonization model. To ensure the safety of daily EcN 2.0 administration under our experimental conditions, we monitored weight gain and evaluated several clinical indicators (Table S3). No significant differences were observed between the two groups for any of these parameters, indicating that the treatment did not cause adverse effects (Table S4).

**Fig. 5:**
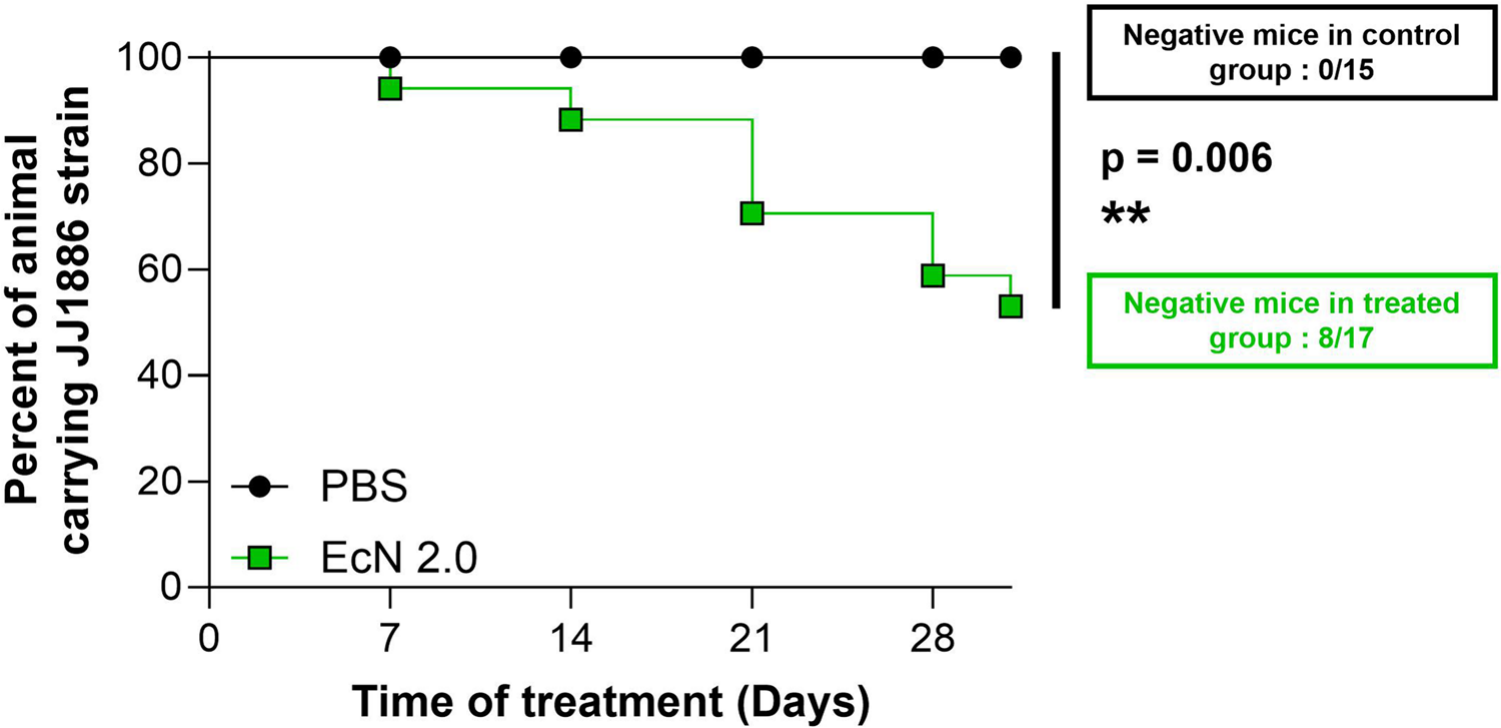
Positive ratio of mice regarding the presence (positive) or absence (negative) of detectable JJ1886 in treated (EcN 2.0) or not (PBS) mice. Mice were considered to be negative for JJ1886 if no colony was found after plating serial dilution of feces on agar plates with the appropriate selection antibiotic (rifampicin; detection threshold = 2 log CFU/g of feces). Mean values ± standard deviation are shown for the two groups: control (untreated mice *n* = 15) or treated (EcN 2.0–treated mice *n* = 17). The difference between the experimental groups was evaluated by the log-rank (Mantel-Cox) test: **p < 0.01

To confirm that the “responders” no longer excrete *E. coli* JJ1886, the results were qualitatively verified (positive versus negative) through the enrichment culture of the feces with the appropriate selection antibiotic (rifampicin 50 µg/mL) in comparison to the “non-responder” animals (Fig. 5). The results confirm the reduced carriage of strain JJ1886 in animals in response to probiotic treatment with EcN 2.0.

### No Microcin Resistance Development in *E. coli* JJ1886 Isolates After Treatment

To gain further insight into the response of the animals to treatment with the probiotic strain EcN 2.0, we sought to elucidate the underlying mechanisms that led to the observed differences between the animals whose bacterial load decreased, designated as “responders,” and those in which the bacterial load remained unaltered, designated as “non-responders.” At the end of the in vivo experiment, colonies of *E. coli* JJ1886 were isolated from feces of animals treated with vehicle or EcN 2.0 and always showed *E. coli* JJ1886 shedding. Those isolates were used to determine if they were still susceptible to the antimicrobial activity of EcN 2.0 in vitro. All isolates had a similar response to EcN 2.0 exposure. The growth limitation of these isolates was comparable to that obtained on the original strain JJ1886, including in non-responding mice (Fig. S4). Strains isolated from PBS group mice showed a similar growth inhibition pattern, compared to *E. coli* JJ1886 isolates in vivo exposed to EcN 2.0 in mice. These results indicate that the JJ1886 isolates from “non-responder” animals in the EcN 2.0-treated group did not acquire microcin resistance in vivo. These results do not support the hypothesis that microcin resistance appeared in *E. coli* JJ1886 in non-responder animals and was selected during the treatment.

### EcN 2.0 Is Efficient Against Human and Animal Strains Isolated from Urinary Tract Infections

Given the increasing emergence of antimicrobial-resistant bacterial strains that cause diseases such as urinary tract infections, the use of bacteria with antibacterial properties as an alternative to antibiotics is being proposed alongside other strategies. To document the potential use of EcN2.0, its antagonistic activity was tested against *E. coli* isolates from urinary tract infections in human patients and pets (dogs and cats). From a bank of 200 *E. coli* isolates collected from patients with bacteriuria (asymptomatic bacteriuria, acute cystitis and pyelonephritis) at the University Hospital of Toulouse [32], several strains were selected, five of which were resistant to at least three families of antibiotics. Similarly, from a bank of 150 *E. coli* isolates collected from dogs and cats at the Veterinary Teaching Hospitals of the Toulouse and Alfort Veterinary Schools, several strains resistant to at least three families of antibiotics were selected [33]. The antimicrobial resistance profile and sequence type are presented in Table S5. EcN WT and EcN 2.0 were tested in liquid co-culture experiments to assess their efficacy to fight selected clinical *E. coli* isolates.

**Fig. 6:**
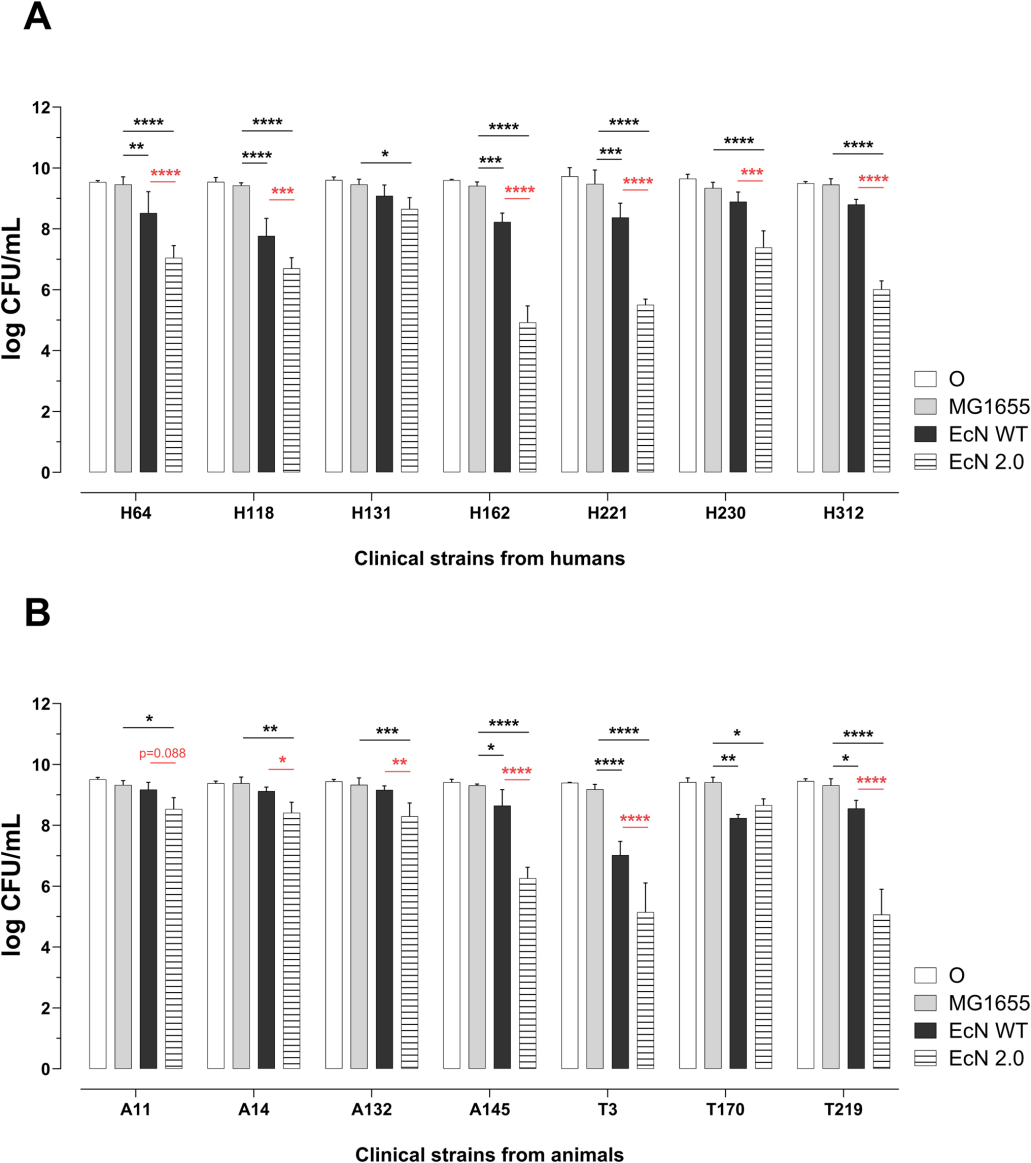
Efficacy of EcN against antimicrobial-resistant clinical strains isolated from humans and pets. Competition in liquid medium. Target clinical strains, human (**A**) and animal (**B**) were grown alone or in combination with an antimicrobially active strain, EcN WT or EcN 2.0 or a negative control (*E. coli* strain MG1655). After 24 h of incubation at 37 °C with agitation, the target strain was enumerated by successive dilutions and spread on antibiotic-enriched agar to select it. *N* = three independent biological replicates. Means values ± standard deviation are shown. Two-way ANOVA with Bonferroni’s multiple comparison post-test. In black, comparison between negative control MG1655 and EcN WT or EcN 2.0. *p < 0.05; **p < 0.01; ***p < 0.001; ****p < 0.0001. In red, comparison between EcN WT and EcN 2.0. *p < 0.05; **p < 0.01; ***p < 0.001; **** *p* < 0.0001

The growth of 4/7 human (H64, H118, H162, and H221) and pet strains (A145, T3, T170, and T219) was inhibited by EcN WT (black asterisk, Fig. 6), reducing the growth by around 1.21 log CFU (mean growth between 7.76 and 8.51 for the four inhibited strains) compared to the exposure of the microcin-negative control strain MG1655 (mean 9.43 log CFU) or clinical strain alone (mean 9.59 log CFU) (Fig. 6A). Similarly in animal strains, around 1.11 log CFU inhibition was observed (mean growth between 7.02 and 8.65 for the four inhibited strains) compared to the negative control MG1655 (mean 9.32 log CFU) or strain alone (mean 9.43 log CFU) (Fig. 6B).

Exposure to EcN 2.0 leads to inhibition of all human and animal strains (black asterisk, Fig. 6). EcN 2.0 had a strong inhibitory effect, reducing growth of human strains by around 3.17 log CFU (mean growth between 4.92 and 7.38 log CFU for the six inhibited strains) compared to the negative control MG1655 (mean 9.43 log CFU) or strain alone (mean 9.59 log CFU) (Fig. 6A). For the animal UTI isolates, the growth of all the strains was significantly reduced with EcN2.0 by approximately 2.12 log CFU inhibition (mean growth between 5.06 and 8.66 log CFU for the seven inhibited strains) compared to the negative control MG1655 (mean 9.32 log) or strain alone (mean 9.43 log) (Fig. 6B).

EcN 2.0 had superior antimicrobial activity than EcN WT on all the strains tested with a difference of 2.16 log CFU (human) and 1.46 log CFU (animal) inhibition between the two probiotic strains tested (red asterisk, Fig. 6). One animal strain (T170) was inhibited at the same level by EcN WT and EcN 2.0. All the five ST131 strains tested were inhibited by EcN2.0 exposure.

## Discussion

The gut is the main reservoir of MDR bacterial strains. To prevent their spread in the environment and to limit the risk of them leaving the digestive tract and causing infections with high morbidity and mortality, decolonization of the digestive tract has advanced as a preventive measure [42].

Classical animal models for the carriage of MDR bacteria are based on the use of antimicrobial treatment and/or a single gavage of the multi-resistant strain of interest to allow its implantation in the animal’s digestive tract. In these dysbiotic models due to the use of antibiotic treatment to install the strain of interest in the digestive tract which introduces a significant bias, the progressive reduction of the bacterial load in the gut also complicates the assessment of the antibacterial effect of the probiotic strains or therapeutic molecules [12, 39, 40]. In this study, the use of the vertical transmission model [41, 43] allows a stable, natural colonization by the MDR *E. coli* bacteria of interest without affecting the development and stability of microbiota in these animals [36, 41, 44]).

In this physiological model with a stable gut microbiota, half of the mice were completely cured of intestinal carriage of the ESBL-producing *E. coli* strain JJ1886, both in feces and in gut tissues, after 1 month of EcN 2.0 treatment. However, nine mice were still colonized with the MDR strain at the end of the experiment. Several hypotheses could be formulated to explain the non-response of these mice. As observed with the use of antibiotics, resistance to microcins M and H47, or more generally to the effects of EcN2.0, could have developed in the *E. coli* JJ1886 colonizing the digestive tract of the mice. However, this hypothesis does not seem plausible because in vitro exposure of *E. coli* JJ1886 clones collected from mice at the end of the treatment with EcN 2.0 showed no difference between responding and incompletely responding individuals. Local iron depletion is also often proposed as a key factor, as it is necessary to observe an adequate microcin production and manifestation of a probiotic effect [45] or a proinflammatory state [46, 47]. Despite the use of an unregulated promoter for the expression of microcins in a plasmid, it could be hypothesized that under our experimental conditions, EcN 2.0 was not able to permanently produce the required amount of microcins M and H47 for stoichiometric reasons, e.g., due to insufficient production of enterobactin to form a functional siderophore microcin complex [19, 48, 49] or other necessary proteins such as TonB or IroN [18]. In addition, the Swiss mice used in the in vivo experiment usually have a high level of genetic diversity [50, 51], which may explain inter-individual differences in response to the treatment with EcN 2.0. In the context of clinical use, the finding of inconsistent efficacy is consistent with clinical reports in human medicine and highlights an individual factor in probiotic efficacy [13–16]. In any case, the ability of EcN 2.0 to complementarily eliminate the stable, natural carriage of *E. coli* JJ1886 in more than half of the treated mice is a very encouraging result for the use of this probiotic in humans or animals with MDR bacteria stably implanted in their gut microbiota.

The permanent colonization of the intestinal microbiome by ExPEC variants can also influence the occurrence of urinary tract infections. UTIs are a widespread disease and the increasing occurrence of (multi-)resistance is also making successful treatment more difficult [52, 53]. Various studies show that recurrent UTIs can also be attributed to repeated smear infections with bacteria from the patient’s intestinal microbiota and that the composition of the intestinal microbiome therefore plays an important role in the occurrence of UTIs (gut-bladder axis) [54, 55]. It is therefore obvious to develop therapeutic approaches that can selectively remove relevant bacteria with an increased potential to trigger UTIs from the microbiome without otherwise triggering dysbiosis of the intestinal microbiome [56, 57].

The fact that the competitive properties of EcN can be exploited to reduce intestinal colonization by intestinal pathogens has already been widely published [17, 58]. In addition, promising data suggest that EcN can also reduce the prevalence of UPEC in the intestinal tract [59]. Against this background and the data generated in our study, the use of EcN 2.0 with its optimized antagonistic potential appears to be very promising for the selective eradication of MDR uropathogens in the intestinal microbiome.

Unlike classical antimicrobial treatment, microcins produced by EcN 2.0 are highly specific and show a narrow spectrum, targeting bacteria belonging to the *Enterobacterales* order, using the same siderophore capture system with enterobactin [60, 61]. As a result, EcN 2.0 could be effective against more problematic multidrug-resistant bacteria defined by the WHO as critical priority, such as third-generation cephalosporin-resistant or carbapenem-resistant *Enterobacteriaceae*, or high priority, such as fluoroquinolone-resistant *Salmonella typhimurium* [62]. Unlike a conventional antibiotic treatment that will impact the entire microbiota, microcins act at low doses, and at a short distance in the digestive tract due to delivery within the same ecological niche of the target bacteria, limiting the risk of perturbation of the microbiota homeostasis [21]. Resistance dissemination is also reduced by the narrow spectrum of action and controlled secretion [22].

In conclusion, our results show that “arming” the probiotic strain EcN with microcin overexpression could be an innovative biotherapeutic tool in the fight against the carriage and spread of MDR and/or ESBL-producing *E. coli* strains. This work is part of the discovery and study of alternative treatments to antibiotics. In the future, it seems essential to develop curative or better preventive treatments against MDR *E. coli* strains, with particular attention to asymptomatic carriers to prevent the dissemination of the resistance determinants.

## Supplementary Information

Below is the link to the electronic supplementary material.

Supplementary file1 (DOCX 504 KB)

## Data Availability

No datasets were generated or analysed during the current study.
