# Supplementary material for: A Non-genotoxic Variant of Escherichia coli Nissle 1917 EcN 2.0 Overexpressing Microcins Reduces Intestinal Carriage of ST131 ESBL-Producing Escherichia coli
Source: Probiotics Antimicrob Proteins. 2025 Nov 11;18(4):5413–27. doi: 10.1007/s12602-025-10777-y (PMC13341728; doi:10.1007/s12602-025-10777-y)
Supplement: Supplementary file 1 — Supplementary file1 (DOCX 504 KB) [file 12602_2025_10777_MOESM1_ESM.docx]

**Supplementary Information**

**A non-genotoxic variant of *Escherichia coli* Nissle 1917 EcN 2.0 overexpressing microcins reduces intestinal carriage of ST131 ESBL-producing *Escherichia coli.***

Nicolas Jousserand^1^, Benjamin Massiera^1^, Pierre-Jean Bordignon^1^, Michelle Boury^1^, Marie Tremblay-Franco^2^, Ulrich Dobrindt^3^, Patricia Martin^1^, Rachel Lavoué^1^, Eric Oswald^1,4,^*^,#^, Delphine Payros^1,^*^,#^

^1^ IRSD, Université de Toulouse, INSERM, INRAE, ENVT, UPS, Toulouse, France.

^2^ Toxalim (Research Centre in Food Toxicology), Université de Toulouse, INRAE, ENVT, INP-Purpan, UPS, Toulouse, France

^3^ Institute of Hygiene, University of Münster, Münster, Germany.

^4^ CHU de Toulouse, Hôpital Purpan, Toulouse, France.

* The authors contributed equally to this work.

Co-corresponding authors: [^#^delphine.payros@inserm.fr](mailto:#delphine.payros@inserm.fr) ; [^#^eric.oswald@inserm.fr](mailto:#eric.oswald@inserm.fr)

**Supplementary Figures 1, 2, 3 & 4**

**Supplementary Table 1, 2, 3, 4 & 5**

**
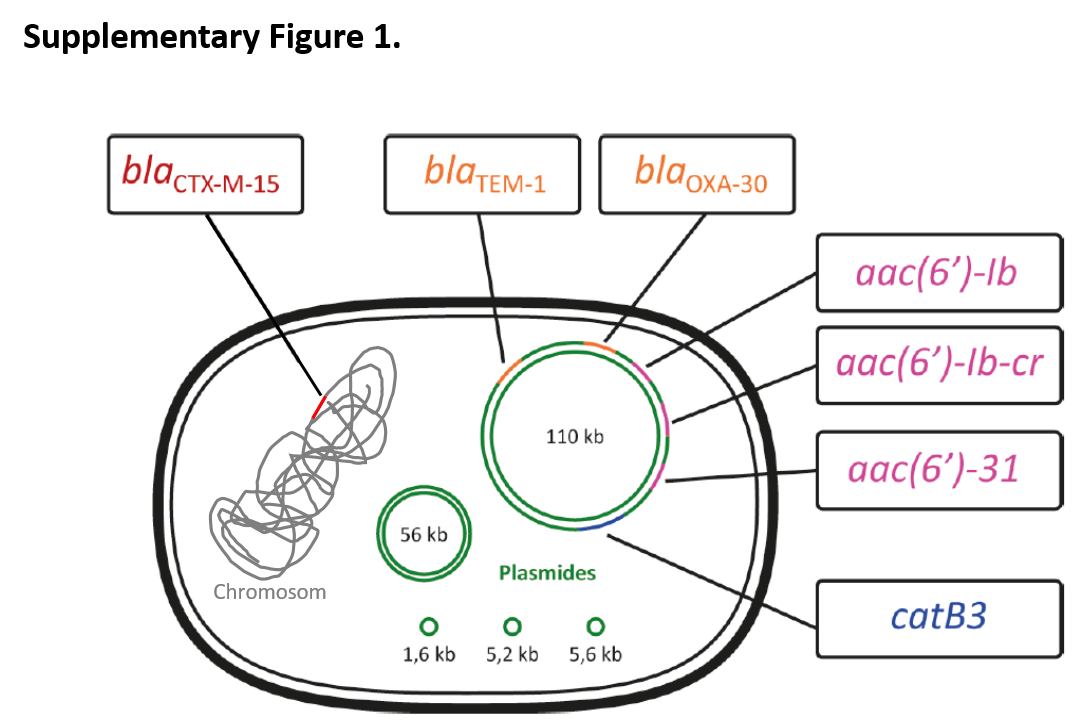
**

**Supplementary Figure 1.**

Schematic representation of the genetic support for antibiotic resistance in strain JJ1886.

Isolated from a patient with recurrent urinary tract infections in 2011, strain JJ1886 belongs to sequence type ST131. As classically found in ST131 isolates, the virulence genes *papA, fimH, iha, fyua, iutA, kpsMII, sat, usp, ompT* and *malX* are present in JJ1886. At the opposite, *papG, sfa/foc, hlyA* and *cnf1* are absent from its genome. The strain carries 5 plasmids (in green), the largest of which carries most of the resistance genes. The bla_CTX-M-15_ gene responsible for ESBL production is integrated into the chromosome. Its antibiogram indicates resistance carried by multiple plasmids: to aminoglycosides and fluoroquinolones, to beta-lactams and to chloramphenicol. The bla_CTX-M-15_ gene is found on the chromosome and codes for cefotaximase ESBLs capable of hydrolyzing 3rd generation cephalosporins. JJ1886 is sensitive to TMPS, piperacillin-tazobactam, carbapenes, fosfomycin and amikacin.

**Supplementary Figure 2.**


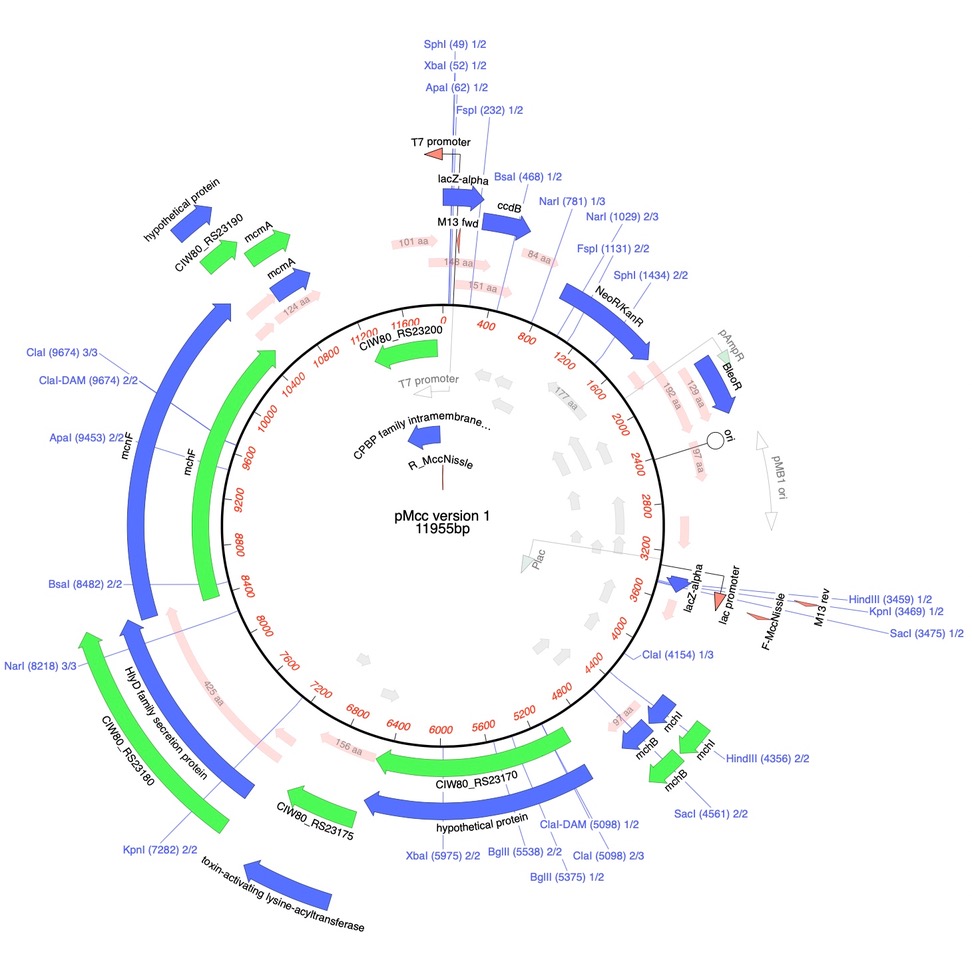


**Supplementary Figure 2.**

Schematic representation of pMcc plasmid, constituted by the commercially-available plasmid TOPO-XL that carries the machinery required for the microcin biosynthesis and export, and the genetic island MccM/MccH47.

**Supplementary Figure 3.**

**Supplementary figure 3.**

Evaluation of gut JJ1886 bacterial shedding in fecal homogenates before the beginning of treatment with PBS (grey bars, control group) or EcN 2.0 (green bars). Each circle represents a mouse.

**Supplementary Figure 4.**

**EcN 2.0 resistance of JJ1886 isolates from mouse feces treated with EcN 2.0 for 31 days, evaluated by competition in liquid medium.** One strain was isolated from feces at the last collecting point (D31) from all the mice with residual JJ1886 shedding. Target strain JJ1886 was grown alone (control) or in combination with an antimicrobial active strain, EcN 2.0, or a negative control (*E. coli* strain MG1655). After 24 hours of incubation at 37°C with agitation, the target strain is enumerated by successive dilutions and spread on antibiotic-enriched agar to select it. One circle represents one strain isolated from one mouse.

Mean values ± standard deviation are shown. One way ANOVA with Tuckey’s multiple comparison post-test. * p < 0,05; *** p < 0,001; **** p< 0,0001.

**Tables**
